# Strategies and enabling conditions for strengthening older adults’ involvement as active research partners: protocol for a sequential mixed-methods study in Sweden

**DOI:** 10.1136/bmjopen-2026-118308

**Published:** 2026-07-20

**Authors:** Ann-Therese Hedqvist, Susanna Strandberg, Charlotta Nilsen, Willemo Carlsson, Roger Carlsson, Paola Violasdotter Nilsson, Mats Holmberg, Linda Ljungholm, Sara Bergstrand, Maria Andreassen, Bodil Holmberg, Joakim Niklasson

**Affiliations:** 1 Department of Health and Caring Sciences, Linnaeus University, Kalmar, Sweden; 2 Department of Ambulance Services, Region Kalmar län, Västervik, Sweden; 3 Department of Health and Caring Sciences, Linnaeus University, Växjö, Sweden; 4 Institute of Gerontology, School of Health and Welfare, Jönköping University, Jönköping, Sweden; 5 Public contributor, Växjö, Sweden; 6 University Library, Jönköping University, Jönköping, Sweden; 7 Department of Health Sciences, Innovation and Design, Mälardalen University, Eskilstuna, Sweden; 8 Department of Health, Medicine and Caring Sciences, Linköping University, Linköping, Sweden; 9 Department of Nursing Science, Sophiahemmet University, Stockholm, Sweden; 10 Applied Health Technology, Blekinge Institute of Technology, Karlskrona, Sweden

**Keywords:** Aged, Delphi Technique, Community-Based Participatory Research

## Abstract

**Abstract:**

**Introduction:**

Older adults are increasingly recognised as valuable contributors in health and social care research, yet their involvement as active research partners remains inconsistent and under-theorised across contexts. The aim of this study is to investigate how older adults are involved as active research partners in health and social care research and to build consensus on strategies and enabling conditions that can support and strengthen such involvement.

**Methods and analysis:**

This study uses a sequential exploratory mixed-methods design comprising three phases. In the preparatory phase, the study protocol was developed and the ethical approval application was prepared and submitted. In the exploratory phase, an umbrella review of international evidence, a mapping survey with older adults and researchers, qualitative interviews and a participatory concept-mapping workshop will be conducted to identify experiences, practices, barriers and strategies for involving older adults as research partners. The empirical components will primarily be conducted within a Swedish context to generate context-sensitive insights. Findings will be triangulated to develop a preliminary framework and candidate consensus statements. In the consensus-building phase, a modified Delphi study will be conducted with two expert panels of older adults and researchers, respectively. Across iterative rounds, participants will rate the importance and feasibility of each statement using 5-point Likert scales. Quantitative data will be analysed descriptively to assess consensus levels and qualitative comments will undergo content analysis. Results will be analysed overall and by panel to identify areas of agreement and divergence.

**Ethics and dissemination:**

The study has received an advisory opinion from the Swedish Ethical Review Authority (reference number 2025–08878-01). The study will be conducted in accordance with Swedish ethical regulations and applicable data protection legislation, including the General Data Protection Regulation. Older adults have been involved in shaping the study and corresponding protocol and will contribute to the interpretation and dissemination of findings. Results of this study will be shared through peer-reviewed publications and conference presentations.

**STRENGTHS AND LIMITATIONS OF THIS STUDY**

- This study addresses a key gap by systematically identifying and establishing consensus on strategies to strengthen the involvement of older adults as active research partners, using a sequential exploratory mixed-methods design conducted in three phases.
- The study integrates multiple complementary data sources—including an umbrella review, a mapping survey, qualitative interviews, a participatory concept-mapping workshop and a Delphi process—providing a robust empirical and conceptual foundation.
- Older adults are actively involved throughout the whole project, including in study design and protocol development, interpretation of findings and as participants in the Delphi expert panel, supporting meaningful and equitable partnership.
- The empirical components are conducted within a Swedish context, which may limit the transferability of findings to other healthcare systems and cultural settings.
- Despite efforts to ensure diversity, participants who choose to engage in the studies may differ systematically from those who do not, which may influence the range of perspectives and representativeness of the consensus.

## Introduction

 Older adults have traditionally been positioned as passive subjects in research, with limited influence over the design, priorities and interpretation of studies in which they are involved.^1^,^5^ In recent years, however, there has been growing recognition of the value of involving older adults not only as participants but also as active partners in research—individuals who contribute meaningfully to various phases of the research process, including agenda-setting, design, data collection, analysis and dissemination.^6^,^8^ This shift reflects the broader evolution of participatory approaches such as co-creation, co-production, citizen science, community-based participatory research and patient and public involvement (PPI), all of which aim to democratise knowledge production and bridge the gap between research and real-world contexts.^19^,^14^ In line with recent calls by the WHO to ‘listen to the voices of diverse groups of older people’ as a prerequisite for equity and healthy ageing, there is a growing imperative to strengthen and support their meaningful involvement as active partners in research.^15^

The term ‘active partners in research’ is used in this study to describe individuals who are engaged in shaping and conducting research in collaboration with professional researchers. These individuals are also referred to in the literature as co-researchers, public contributors or PPI partners, depending on disciplinary and regional contexts.^19^,^13 16^ At the same time, the understanding and implementation of such participatory approaches vary across countries and research traditions, influenced by differences in policy frameworks, cultural norms and expectations regarding the roles of patients and the public in research.

Involving older adults as active partners in research has been associated with a range of benefits. Research shows that such engagement can improve the quality, relevance and applicability of findings by ensuring that research questions, measures and interventions are grounded in the lived experiences, priorities and contextual knowledge of the populations they aim to serve.^17^,^21^ Older adults can help refine terminology, identify overlooked needs, highlight practical barriers and strengthen the cultural and contextual fit of study designs, which in turn can enhance methodological rigour and real-world applicability. Their involvement can also increase transparency and ethical robustness by ensuring that study procedures and outputs align with participants’ values and expectations. Beyond these direct contributions to research design and interpretation, involvement may enhance community trust, counter ageist stereotypes and empower individuals through skill-building, recognition and shared ownership of the research process.^1022^,^27^ These relational and social mechanisms can further strengthen the relevance and uptake of research findings in practice. However, these benefits are not uniformly realised across settings, and their extent may depend on contextual factors such as institutional support, research culture and the availability of structures for sustained engagement.

Further positive outcomes have been revealed by systematic reviews that show increased social inclusion, civic participation and the development of more acceptable and sustainable interventions.^28^,^30^ However, despite these advances, significant challenges remain. The meaningful involvement of older adults is often constrained by power imbalances, tokenistic practices, limited recruitment strategies and the lack of structures for sustained engagement throughout the research process.^31^,^34^ In Nordic countries, although interest in PPI and related approaches is growing, the field remains underdeveloped, with unclear expectations and limited policy mandates for involving older adults as partners in research.^35 36^

To begin addressing these knowledge and practice gaps, the study will first synthesise existing international evidence on the involvement of older adults as active research partners through an umbrella review of systematic reviews. This review will map current definitions, conceptual frameworks, reported benefits and challenges and documented strategies for involvement across different contexts, thereby establishing a broad conceptual foundation for the subsequent phases of the study.

Building on the insights from the umbrella review, the study will explore contemporary practices, experiences and stakeholder perspectives using a sequential exploratory mixed-methods design. The empirical phases of the study, including the mapping survey, qualitative interviews and concept-mapping workshop, will primarily be conducted within a Swedish context. Through these components, the study will identify key concepts, barriers, enablers and potential strategies for strengthening older adults’ involvement in research. These findings will inform the development of statements for a modified Delphi process, through which older adults and researchers will collaboratively establish consensus on principles and strategies to support meaningful partnership. As a whole, the study aims to generate evidence that can inform future policy, education and practice to promote more inclusive and collaborative research in older populations. Although the findings are context-sensitive, they are expected to provide transferable insights relevant to similar research and healthcare settings.

### Aims and study objectives

The aim of this study is to explore how older adults are involved as active research partners in health and social care research and to build consensus on strategies and enabling conditions that can support and strengthen such involvement.

The specific objectives are:

1. To explore current practices, experiences and contextual factors related to the involvement of older adults as active research partners in health and social care research.
2. To identify, assess and prioritise strategies and conditions that can enhance and support the involvement of older adults as active research partners.

### Definition of key terms

In this study, the lower age limit for defining ‘older adults’ is set at 60 years, in line with the WHO’s classification, which commonly uses 60 years as the threshold for older age in global health and ageing research.^15 37^ This definition ensures international comparability while acknowledging that chronological age is an imperfect proxy for ageing. The experience of being older is highly individual, as ageing is shaped by genetics, lifestyle, health status and social factors.^15 37 38^ Accordingly, the review adopts an inclusive approach, recognising both the heterogeneity of older populations and the diversity of ageing experiences across contexts.

## Methods and analysis

### Study design

**Figure 1:**
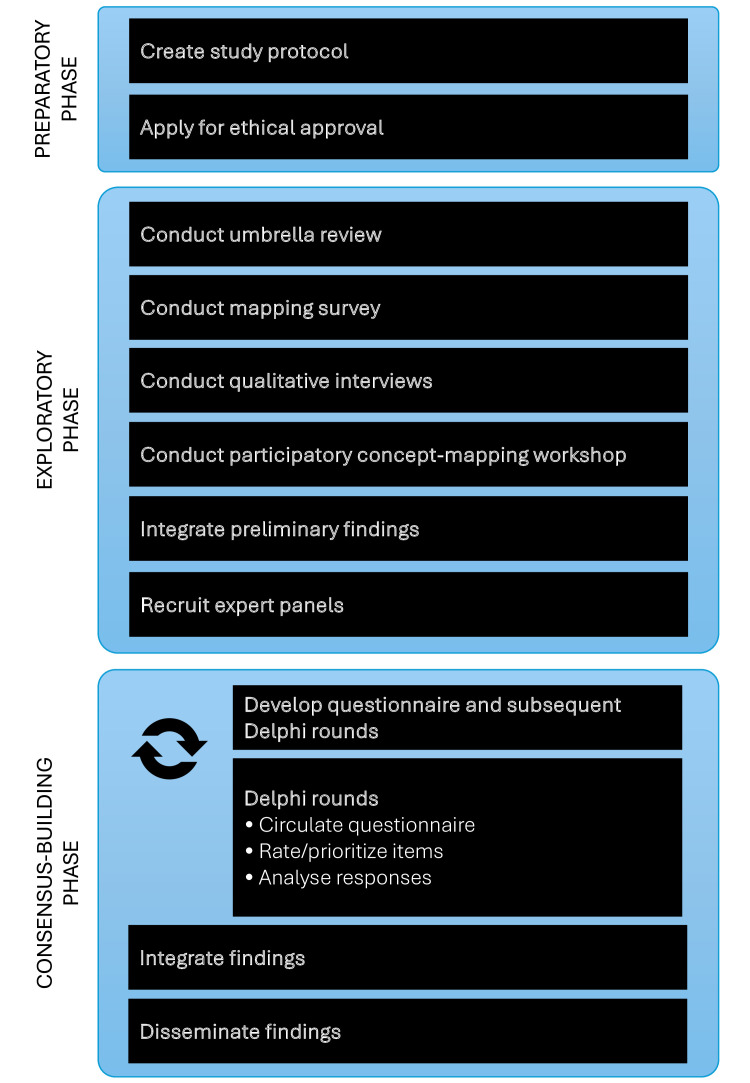
Overview of the sequential exploratory mixed-methods study design consisting of a preparatory phase, an exploratory phase and a consensus-building phase.

This study follows a sequential exploratory mixed-methods design^39^ consisting of three phases: (1) a preparatory phase, (2) an exploratory phase and (3) a consensus-building phase (figure 1). The design is based on participatory principles and evidence-informed methodology^40^ to ensure meaningful involvement of older adults and other stakeholders throughout the research process. This protocol is reported in accordance with the Standard Protocol Items: Recommendations for Interventional Trials (SPIRIT) 2013 statement.^41^

The research team comprises researchers with expertise in healthcare research, ageing, participatory research and mixed-methods design, including representation from multiple healthcare professions, as well as a senior academic librarian with expertise in systematic literature searching and review methodology, and two older adults with lived experience of involvement in research processes. The team was assembled to ensure complementary methodological, clinical and experiential perspectives, supporting both scientific rigour and meaningful involvement throughout the study.

The study commenced in November 2025, with data collection planned between January 2026 and December 2027. At the time of submission, the umbrella review has been initiated and recruitment for subsequent study phases is ongoing. No final analyses have been conducted. The protocol has been prospectively registered in the Open Science Framework (OSF) (DOI: 10.17605/OSF.IO/TPMX9).

#### Phase 1: preparatory phase

The preparatory phase focuses on establishing the methodological, ethical and logistical structure for the study. This includes the development of the study protocol and the preparation and submission of the ethical approval application. No empirical data are collected during this phase.

#### Phase 2: exploratory phase

The exploratory phase comprises four substudies designed to build an empirical and conceptual foundation for the subsequent Delphi process. First, an umbrella review synthesises existing evidence on older adults’ involvement as research partners.^42^ Whereas the umbrella review synthesises international evidence, the subsequent empirical phases are conducted primarily within a Swedish context.

Second, a mapping survey is distributed to a broad sample of older adults and researchers to capture involvement practices, perceived challenges, enabling conditions and priorities related to involvement in research. The survey provides an overview of the prevalence and variation of experiences across stakeholder groups and identifies areas requiring deeper exploration.

Third, qualitative interviews with older adults and researchers are conducted following completion of the survey. The interviews build directly on the survey findings and are designed to deepen understanding of participants’ experiences, interpretations and contextual reasoning underlying the patterns identified in the quantitative data.^43^

Fourth, a participatory concept-mapping workshop will be conducted at an international conference with older adult representatives, researchers, healthcare professionals and other stakeholders. Following established concept-mapping methodology,^44^ participants will collaboratively generate, sort and structure ideas related to barriers, enablers and strategies for involvement.

Across these four exploratory components, findings will be iteratively integrated to inform subsequent data collection and analysis. This includes preliminary integration during the exploratory phase to guide the development of the survey, interview guide and workshop design. The final triangulation will result in a preliminary framework and an initial pool of statements for the subsequent consensus-building phase.

#### Phase 3: consensus-building phase

The third phase consists of a modified Delphi process^45 46^ conducted with two expert panels: older adults and researchers, respectively. The Delphi will involve multiple iterative rounds (typically two to three, but flexible as needed) in which participants rate and comment on statements derived from Phase 2. Controlled feedback will be provided between rounds to support convergence of opinions. The aim of this phase is to establish consensus on key strategies, values and enabling conditions necessary for meaningful involvement of older adults as active research partners.

### Participants and recruitment

This study will involve two main participant groups: (1) older adults (aged 60 years and above) and (2) researchers.

Participants in the empirical phases will primarily be recruited within Sweden. Inclusion criteria for older adults are age 60 years or older and previous experience of, or interest in, contributing to research as active partners. Inclusion criteria for researchers are experience in participatory research approaches and/or in health, social care or ageing-related research, including involvement of older adults as research partners. Participants must be able to understand and communicate in Swedish or English. No formal exclusion criteria are applied beyond these requirements.

Older adults will be recruited through civil society organisations, community networks, PPI registries and targeted outreach in relevant forums. The aim is to ensure diversity in age, gender, health status and previous experience of research involvement. Researchers will be purposively sampled based on their experience with participatory approaches such as co-design, co-production or community-based participatory research in health and social care. Recruitment will be conducted through academic institutions, conferences, professional networks and research centres focused on ageing, public involvement and collaborative methodologies.

The mapping survey will be distributed to a broader purposive sample, with the aim of achieving a final sample of 100–200 respondents. To reach this target, the survey will be disseminated to a larger pool of potential participants. Efforts will be made to obtain a reasonably balanced representation of older adults and researchers, aiming for 50–100 participants in each group. This sample size is appropriate for the exploratory purpose of the survey and is expected to allow identification of patterns in current involvement practices, as well as perceived barriers and enabling factors. Participants for the qualitative interviews may be recruited among survey respondents who indicate interest in further participation, as well as through additional purposive sampling to ensure variation in experiences and perspectives.

For the qualitative interviews, approximately 20–25 participants—including both older adults and researchers—will be recruited to ensure variation in experiences and perspectives. The sample size is based on the concept of information power,^47^ meaning that the final number will be determined by the richness and relevance of the data rather than by a fixed target. Recruitment will continue until the data are sufficiently informative to support robust thematic development.

Participants for the concept-mapping workshop will be recruited during an international conference where the workshop is hosted as an open session. Stakeholders anticipated to attend include older adults, PPI representatives (ie, individuals with prior experience of PPI in research, including advisory or co-research roles), researchers, healthcare professionals and policymakers. Attendance will be voluntary; interested participants will receive brief written information about the study and provide informed consent before contributing to the workshop activities. To support diversity of perspectives, participants will be invited to self-identify their stakeholder role (eg, older adult, researcher, PPI representative, clinician, policymaker). The workshop will be conducted as a single in-person session, facilitated by the lead researcher (A-TH) and one coauthor (SS). To reduce the risk of over-representation from any one stakeholder group (eg, a higher proportion of researchers relative to older adults), participants will be distributed across tables to achieve mixed stakeholder groups. Each group will have approximately four to seven participants, with older adults, researchers and other stakeholder types represented within each group wherever feasible. This arrangement supports balanced participation and enables the integration of diverse viewpoints during idea generation, sorting and clustering activities.

Two expert panels will be recruited for the Delphi study: one consisting of older adults (aged 60+ years) and one consisting of researchers with relevant experience in ageing research, participatory research or PPI. Participants in the Delphi panels may partially overlap with participants from earlier phases, with additional participants recruited to ensure broader representation.

Each panel will aim for 15–25 participants, which aligns with recommended panel sizes for Delphi studies in health research and is sufficient to ensure diversity while maintaining manageability across rounds and reduce the risk of dropouts. Recruitment will be informed by principles of expertise, diversity of perspectives and practical feasibility. Older adult panel members will be recruited from networks involved in earlier study phases (survey, interviews, workshop) as well as external organisations. Researcher panel members will be recruited through academic networks, research centres and professional associations. Expression-of-interest forms will be distributed in earlier phases to identify potential participants. Participants will provide consent prior to Delphi round 1.

**Figure 2:**
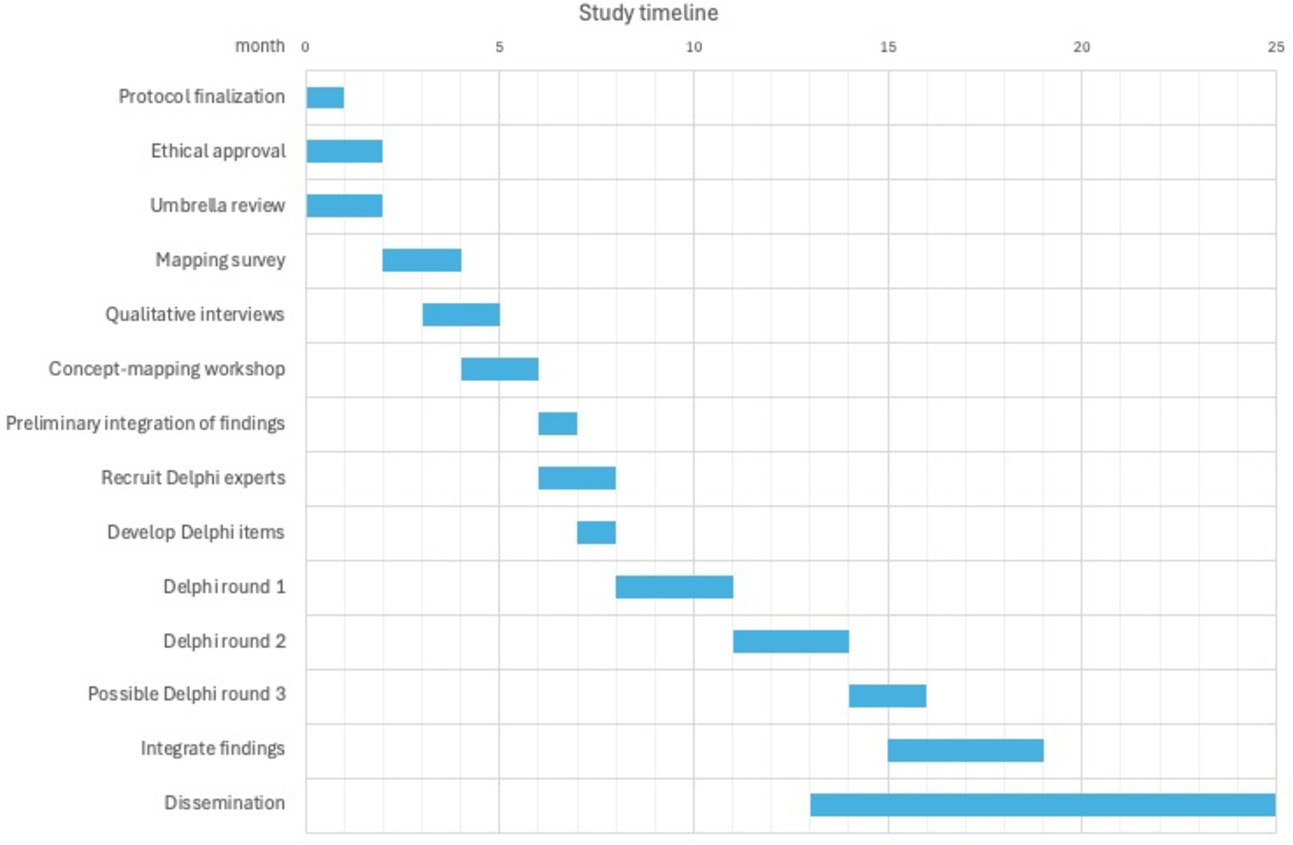
Study timeline showing the sequencing of all study components. Numbers represent project months (0–25).

A study timeline is presented in figure 2, illustrating the sequencing and timing of all study components.

### Data collection

#### Umbrella review

As part of the preparatory phase, an umbrella review will be conducted following the Joanna Briggs Institute methodology for umbrella reviews.^42^ The review aims to synthesise review-level evidence on the involvement of older adults as active research partners in health and social care research. Key data to be extracted include definitions, conceptual models, reported benefits and challenges and contextual factors influencing involvement. The protocol for the umbrella review is registered with PROSPERO (ID: CRD420251064947) and reported separately.^48^ The findings from the umbrella review will provide a conceptual and empirical foundation for the subsequent phases of the study. Specifically, they will inform the development of the semi-structured interview guide by identifying key themes, gaps and contextual factors related to the involvement of older adults in research.

#### Mapping survey

Data for the mapping survey will be collected through structured questionnaires administered to older adults (aged 60+ years) and researchers (see onlinesupplemental files 1  2). The survey is designed to capture current practices, experiences, perceived barriers and enabling conditions related to involving older adults as research partners and includes three components for both groups: demographic variables, background characteristics and key outcome measures. All instruments included are available in validated Swedish versions.

For older adult participants, demographic data will include age, gender, educational level, living situation and self-perceived economic situation. Attitudes toward technology will be assessed using the TechPH scale,^49^ acknowledging that the survey is conducted partly online and in a context where digital technologies permeate everyday life. Technology readiness is therefore considered a critical factor influencing individuals’ ability and willingness to participate in research as active partners. Attitudes toward ageing will be assessed using the Attitude Toward Own Ageing subscale from the Swedish version of the Philadelphia Geriatric Center Morale Scale.^50^ Subjective perceptions of ageing are considered relevant to individuals’ willingness and perceived ability to participate as active research partners, and this measure has shown satisfactory psychometric properties in a Swedish context while also being widely used in subjective ageing research across different age groups.^50 51^ Key outcome measures will focus on previous experiences of participating in research or being invited to do so, perceived barriers and facilitators captured through open-ended items, and preferences for future involvement as research partners.

For researchers, demographic variables will include research field, years of research experience, institutional affiliation and prior experience of involving older adults in research. Organisational and structural conditions—such as institutional support, access to funding and training in engagement methods—will be assessed, as these factors may influence researchers’ capacity to involve older adults as research partners. Key outcome measures will include current engagement practices, reflections on meaningful involvement and perceived barriers and enabling factors, in addition to open-ended items on needs for future support and capacity-building.

The survey will be distributed primarily online via a secure platform, with the option for participants to receive and return a paper version by post if preferred. The survey will be available in Swedish and English.

#### Qualitative interviews

Qualitative semi-structured interviews^43^ will be conducted to explore experiences, perceptions and contextual factors related to the involvement of older adults as active research partners in health and social care research. The purpose of the interviews is to deepen the understanding of current practices and to inform the development of relevant and context-sensitive items for the participatory concept-mapping workshop and subsequent Delphi process. Participants will be selected based on prior experience of involvement in research as partners or collaboration with such partners, to ensure relevance to the study aim. Preliminary semi-structured interview guides for older adults and researchers have been developed based on emerging insights from the ongoing umbrella review and will be refined iteratively as the review progresses (see onlinesupplemental files 3  4). Key topics include participants’ experiences of involvement processes, perceived barriers and enablers, contextual considerations, and suggestions for strengthening future involvement of older adults in research.

Interviews will be carried out by members of the research team with experience in qualitative methods. Interviews will be conducted individually, either in person, by phone or via video call, depending on participant preference and logistical considerations. All interviews will be audio-recorded with consent and transcribed verbatim. Field notes may be used to capture contextual impressions.

#### Participatory concept-mapping workshop

Data will be collected during an in-person concept-mapping workshop^44^ conducted at an international conference. Participants (eg, older adults, PPI contributors, researchers, healthcare professionals, policy actors) will generate and collaboratively sort ideas concerning strategies and enabling conditions for involving older adults as active research partners. Data sources will include written idea statements (post-it notes, worksheets), clustered maps and group-generated categories (photographed and archived), plenary discussion notes, facilitator field notes and demographic information (age group, gender, country, role). All data will be group-generated, non-sensitive and pseudo-anonymised at the point of collection. No personal identifiers will be recorded. Workshop outputs will be analysed using qualitative clustering and thematic synthesis and will inform the development of Delphi statements in the next phase.

#### Modified Delphi rounds

The consensus-building phase will involve a modified Delphi process with two expert panels.^46^ Panel members will be selected from survey respondents who express interest and meet the eligibility criteria. In the first round, participants will be presented with draft consensus statements derived from the umbrella review, mapping survey, qualitative interviews and workshop and asked to rate each statement’s importance and feasibility on 5-point Likert scales (1=not at all important/feasible; 5=extremely important/feasible). Participants will also have the opportunity to provide open-ended comments and suggest additional statements they consider important but missing from the list. In the following rounds, participants will receive summarised group-level feedback from the first round and be asked to review and, if desired, revise their ratings.^52^ All rounds will be conducted online, with the option for participants to complete the questionnaires on paper and return them by post if they prefer.

### Data analysis

This study applies a mixed-methods analytical approach in line with the sequential exploratory design.^39^ Qualitative and quantitative data will be analysed separately using appropriate methods and then integrated to generate a comprehensive understanding of current practices, perceived barriers and enablers, and consensus-based strategies for involving older adults as research partners. Given the context-sensitive nature of the empirical phases, analyses will take into account how findings are shaped by the Swedish research and healthcare context.

#### Analysis of mapping survey

Quantitative data from the mapping survey will be analysed using descriptive statistics to summarise participant characteristics, reported experiences and current practices related to the involvement of older adults as active research partners. Categorical variables will be presented as frequencies and percentages, and continuous variables as means and SD or medians and IQRs, depending on distribution. Distributional assumptions will be checked prior to selecting summary measures. Scale-based measures, including TechPH scores and Attitude Toward Own Ageing, will be summarised in accordance with their respective scoring guidelines, to characterise attitudes toward technology, attitudes toward own ageing and perceived barriers and facilitators to involvement. Inferential statistics will be used to explore associations between participant characteristics and key outcomes. Depending on variable type and distribution, appropriate statistical tests (eg, t-tests, χ^2^ tests, Mann-Whitney U tests or analyses of variance/Kruskal-Wallis tests) will be applied to compare groups. Given the exploratory nature of the study, inferential analyses will be interpreted cautiously and used primarily to identify patterns rather than to establish causal relationships.

Qualitative data from open-ended survey items will be analysed using content analysis^53^ to identify recurring categories, contextual nuances and unique perspectives across respondent groups. Coding will be conducted inductively by at least two members of the research team, who will compare and refine coding categories through an iterative process; any discrepancies will be resolved through discussion until consensus is reached.

#### Thematic analysis of qualitative interviews

Interview data will be analysed using thematic analysis following the six-step approach described by Braun and Clarke.^54^ The process includes: (1) familiarisation with the data; (2) generating initial codes; (3) searching for themes; (4) reviewing themes; (5) defining and naming themes; and (6) producing the report.

In the first phase, transcripts will be read repeatedly so the researchers gain familiarity with the content and can identify meaningful patterns related to older adults’ involvement in research. Initial codes will then be generated systematically across the dataset to label features of interest. These codes will be sorted into potential themes, gathering all relevant data for each theme. Next, themes will be reviewed in relation to both the coded extracts and the full dataset, to ensure internal consistency and distinctiveness. Themes will then be clearly defined and named to reflect their core meaning. Lastly, the results will be written up in a clear and structured way, including illustrative quotes from the interviews.

To support the organisation and coding of the data, NVivo or equivalent qualitative analysis software will be used. To ensure trustworthiness, parts of the material will be coded independently by at least two researchers, and any discrepancies will be discussed until consensus is reached. The thematic findings will guide the design of the participatory concept-mapping workshop and inform the development of initial Delphi statements, ensuring that the perspectives and experiences of both older adults and researchers are well-represented in the next phases of the study.

#### Analysis of participatory concept-mapping workshop

Data from the participatory concept-mapping workshop will be analysed using an inductive, structured mapping approach grounded in established concept-mapping methodology.^44^ Analysis will involve three steps. First, all idea statements generated during the workshop will be consolidated into a single dataset, cleaned for duplicates and grouped into a refined list of unique items. Second, participant-generated clusters (documented through photographs and notes) will be reconstructed in NVivo or equivalent software and compared across groups to identify recurring patterns. These clusters will be used to develop preliminary thematic categories that reflect how participants conceptualised barriers, enablers, values and strategies for involving older adults as research partners. Lastly, plenary discussion notes and facilitator observations will be integrated to refine theme labels and clarify areas of convergence and divergence. The resulting thematic structure will be synthesised with findings from the umbrella review, mapping survey and interviews to support triangulation and inform the development of candidate Delphi statements. All data are group-generated and pseudo-anonymised; no individual-level coding will be performed.

#### Analysis of rounds in modified Delphi study

Ratings from the rounds of the modified Delphi study will be analysed both across the full sample and separately for each expert panel. Descriptive statistics (mean, IQRs and percentage agreement) will be used to assess levels of consensus regarding the importance and feasibility of each statement. Statements reaching predefined thresholds for high agreement (eg, median ≥4 with IQR ≤1) will be considered to reflect consensus. Comparison between panels will be performed to identify both shared priorities and areas of divergence. Free-text comments provided during the Delphi rounds will undergo qualitative content analysis to support the interpretation of rating patterns and to refine or clarify statements between rounds if needed.

#### Integration of findings

In the final stage of the study, findings from the umbrella review, mapping survey, qualitative interviews, participatory concept-mapping workshop and modified Delphi study will be synthesised to generate an integrated understanding of strategies and conditions that support meaningful involvement of older adults as research partners. This triangulation of evidence will highlight converging insights across methods and stakeholder groups and inform the development of practical recommendations and models for future implementation.

### Patient and public involvement

Older adults (WC and RC) have been actively involved as public contributors from the earliest stages of this study. Their lived experience has informed the development of the research questions, the use of inclusive terminology and the overall framing of the study. Although their insights enhance the relevance of the study, we recognise that their contributions are shaped by their own backgrounds and experiences. To address this, we engaged in reflexive discussions within the research team and ensured that decisions were informed by the broader evidence base rather than by individual perspectives alone. The protocol was reviewed by the public contributors to improve clarity and accessibility, with attention to the possibility that their familiarity with research or personal motivations could influence interpretation. Their involvement will continue throughout the whole project, including in the interpretation of findings and the co-creation of dissemination materials. This ongoing engagement will incorporate structured reflexive dialogue to examine how contributors’ and researchers’ assumptions may shape analytical choices. Their contributions are grounded in principles of meaningful involvement and co-production, with deliberate attention to transparency and shared decision-making. Public contributors are recognised as coauthors of this protocol and are affiliated as independent contributors from Sweden.

## Ethics and dissemination

This study will be conducted in accordance with the principles outlined in the Declaration of Helsinki. An advisory opinion has been obtained from the Swedish Ethical Review Authority (reference number 2025–08878-01) prior to the recruitment of participants and data collection. Informed consent, the right to withdraw and voluntary participation will be ensured for all participants. Special attention will be given to the autonomy, capacity and comfort of older adult participants, with procedures adapted as needed to support meaningful and equitable engagement. Confidentiality and data protection will be maintained in accordance with the General Data Protection Regulation. All data will be pseudonymised and stored securely. No personally identifiable information will be published.

The reporting of this study will follow established guidance from the Enhancing the QUAlity and Transparency Of health Research (EQUATOR) Network, including Preferred Reporting Items for Systematic Reviews and Meta-Analyses (PRISMA)^55^ for the umbrella review, the Strengthening the Reporting of Observational Studies in Epidemiology (STROBE)^56^ for the survey and workshop and Conducting and REporting of DElphi Studies (CREDES)^57^ for the Delphi study. Reporting of the qualitative components of the study, including interviews and open-ended survey responses, will follow the Consolidated Criteria for Reporting Qualitative Research (COREQ) checklist.^58^ The mixed-methods integration will be described in line with Good Reporting of A Mixed Methods Study (GRAMMS),^59^ and future publications from the study will adhere to those standards. Any substantial amendments to the protocol will be updated in the Open Science Framework registration and communicated in future publications.

The dissemination strategy is designed to share findings from all phases of the study with academic, professional, policy and public audiences, including older adults. Outputs will include multiple open-access, peer-reviewed publications reflecting the different methodological strands of the study, such as the evidence synthesis, the qualitative and survey-based components, the participatory concept-mapping workshop and the Delphi consensus process. Together, these publications will provide complementary perspectives on strategies, principles and enabling conditions for involving older adults as active research partners. In addition to academic articles, findings will be disseminated through conference presentations, workshops, policy briefs and targeted communication to organisations representing older adults and PPI communities. Older adult partners will be actively involved in shaping accessible summaries for broader public and community dissemination. All outputs will be made open access wherever possible to maximise reach, transparency and societal impact.

## Discussion

This study addresses a methodological and ethical gap in health and social care research by promoting the active involvement of older adults as research partners. Although older adults are increasingly recognised as valuable contributors to research, they are still more often included as study participants than as active co-creators in shaping research processes and outcomes. This limits the potential to harness their lived experiences in ways that improve relevance, inclusivity and real-world applicability.^2 60 61^ Despite growing momentum in participatory approaches, challenges such as power imbalances, tokenism, unclear definitions of involvement and insufficient long-term structures for engagement continue to constrain meaningful participation.^62^,^68^ The present study seeks to address these challenges by identifying enabling conditions for genuine participation through the perspectives of both older adults and researchers.

The study also responds to calls for greater transparency and methodological rigour in research involving public contributors.^69^ An important feature of the modified Delphi design is its preservation of participant anonymity, which reduces hierarchical influence and supports more reflective contribution.^70^,^72^ At the same time, involving older adults as research partners raises ethical and epistemological challenges. Unlike study participants, research partners may be expected to share personal experiences publicly, navigate institutional norms and engage with unfamiliar academic frameworks.^73 74^ Moreover, experiential knowledge may sometimes be granted interpretive authority without being subjected to the same critical scrutiny as scientific analysis, and individual contributors may not necessarily represent the broader groups whose perspectives they are assumed to reflect.^75 76^ These challenges underline the need for clearly defined roles, transparent expectations and continuous reflection on how different forms of knowledge are balanced in the research process.^77 78^

A further challenge concerns representation and inclusivity. Co-research initiatives often attract participants who are already confident, resourceful or familiar with engagement processes, which risks narrowing the diversity of perspectives represented.^79^ To mitigate this, the study incorporates deliberate strategies to broaden participation, including recruitment beyond established networks and the use of accessible formats and facilitation to support contributors with varying capacities. Meaningful involvement depends not only on creating opportunities to participate, but also on critically examining who remains excluded and how participation structures may privilege certain voices over others.^80^

Lastly, the study contributes methodologically by combining multiple qualitative and quantitative approaches to generate both breadth and depth of insight. Aguayo *et al*^81^ emphasise that meaningful involvement requires structured, transparent and user-centred methods that reduce barriers and support contributors effectively—particularly in complex or digitally mediated research environments. By integrating an umbrella review, survey, interviews, participatory concept mapping and Delphi consensus rounds, the present study aligns with these recommendations and enables triangulation across methods and stakeholder groups. In doing so, the study aims not only to generate findings, but also to suggest a transparent and reproducible process for involving older adults as active research partners.

### Expected contribution

The study is expected to generate empirically and consensus-informed strategies to strengthen the role of older adults as active partners in research. In doing so, it will support the development of more inclusive, ethically sound and methodologically robust research practices and contribute to ongoing efforts to democratise knowledge production in health and social care.

The work builds on the argument advanced by Walker,^2^ who emphasised that involving older adults in research is not only a matter of quality—ensuring research relevance and contextual grounding—but also a matter of rights. Older people have historically faced age-based discrimination and social exclusion; engaging them meaningfully in research is a step toward addressing these injustices. The involvement of older adults is therefore an ethical imperative as much as a scientific one.^82^ The present study, in line with the WHO’s Decade of Healthy Ageing,^15^ answers the call to ‘listen to the voices of diverse groups of older people’ to ensure equity and promote opportunities for ageing well.

However, the study has several anticipated limitations. First, the empirical components are conducted within a Swedish context, which may influence the transferability of findings to other healthcare systems and cultural settings. Second, despite efforts to broaden recruitment, there is a risk that participants—both researchers and older adults—may represent individuals already positively inclined toward involvement, potentially limiting the diversity of perspectives. Third, as this is an exploratory mixed-methods study, the findings will primarily generate insights into practices, perceptions and consensus rather than establish causal relationships. These limitations will be considered when interpreting and disseminating the results.

By addressing both methodological and ethical dimensions of involvement, the study contributes to advancing more inclusive and sustainable models of co-produced research in health and social care.

## Supplementary material

10.1136/bmjopen-2026-118308 online supplemental file 1

10.1136/bmjopen-2026-118308 online supplemental file 2

10.1136/bmjopen-2026-118308 online supplemental file 3

10.1136/bmjopen-2026-118308 online supplemental file 4
