# Supplementary material for: Strategies and enabling conditions for strengthening older adults’ involvement as active research partners: protocol for a sequential mixed-methods study in Sweden
Source: BMJ Open. 2026 Jul 20;16(7):e118308. doi: 10.1136/bmjopen-2026-118308 (PMC13386054; doi:10.1136/bmjopen-2026-118308)
Supplement: online supplemental file 1 [file bmjopen-16-7-s001.pdf]

Older Adults as Research Partners

Appendix-ready English version of the older adult questionnaire used in the study

Information and consent. Purpose: We want to understand how older adults (60+) view being involved in research—not only as participants, but also as research partners (co-researchers) who can influence the planning, conduct, and dissemination of research.

What does it mean to be a research partner? It can, for example, involve contributing views on research questions and study design; helping to develop interview questions, questionnaires, or information materials; participating in workshops or reference groups; interpreting results together with researchers; and contributing to the dissemination of results (e.g., lectures, popular science communication, or advice to organizations).

Voluntariness and confidentiality: Your participation is voluntary. You may withdraw at any time. Your responses will be handled confidentially and reported only at group level.

A link to the full study information sheet is provided in the original survey.

Estimated completion time: approximately 15–20 minutes.

The original questionnaire was administered in Swedish. This English version has been formatted as a clean publication appendix.

1 **I have read the information above and consent to participate.** [ ] Yes

Background

2 **Gender**

[ ] Male
[ ] Female
[ ] Other
[ ] Prefer not to say

3 **Age (in years)**

____

4 **Country/region of birth**

[ ] Sweden
[ ] Other Nordic countries and Europe
[ ] Middle East and North Africa
[ ] Sub-Saharan Africa
[ ] Asia and Oceania
[ ] North and South America

5 **Highest completed education**
[ ] Did not complete primary school
[ ] Elementary school
[ ] Primary school / compulsory school
[ ] Upper secondary school
[ ] Vocational qualification
[ ] University / college, 1–3 years
[ ] University / college, more than 3 years
[ ] Doctoral education

6 **Civil status**
[ ] Married
[ ] Cohabiting
[ ] Living apart together (LAT)
[ ] Single
[ ] Widow / widower
[ ] Prefer not to say

7 **Housing type**
[ ] Detached house
[ ] Owner-occupied apartment / condominium
[ ] Rental apartment
[ ] Sheltered housing
[ ] Rental apartment in special housing (e.g., residential care home)[ ] Other

8 **Household size (including yourself)**[ ] 1
person
[ ] 2 persons
[ ] 3 persons
[ ] More than 3 persons

9 **How often do you participate in organizations/associations or volunteer work?**
[ ] Never
[ ] A few times per year
[ ] A few times per month
[ ] A few times per week
[ ] Several times per week

Experience of Research Participation and Research Partnership

10 **Have you previously participated in research (e.g., completed a questionnaire, been interviewed, or taken part in a clinical study)?**
[ ] Yes
[ ] No
[ ] Do not know

11 **Have you ever been asked to be a research partner / co-researcher (e.g., member of a reference group, co-creator, or adviser)?**

[ ] Yes
[ ] No
[ ] Do not know

12 **If yes: In what type of role have you been involved? (Select all that apply)**[ ] Reference group / user advisory council
[ ] Workshop / co-design
[ ] Participation in study planning (purpose, questions, design)
[ ] Helped develop information materials / questionnaire / interview guide
[ ] Contributed to the interpretation of results
[ ] Contributed to the dissemination of results
[ ] Other, please specify

13 **How did you experience your involvement as a research partner?**

[ ] Very positive
[ ] Fairly positive
[ ] Neither positive nor negative
[ ] Fairly negative
[ ] Very negative
[ ] Not applicable

14 **Please tell us more about how you experienced your involvement as a research partner.**

15 **Did you feel that you received sufficient support (e.g., information, time, compensation, clarity about the role)?**

[ ] Yes, completely
[ ] Yes, partly
[ ] No
[ ] Do not know
[ ] Not applicable

16 **Please tell us more about how you experienced the adequacy of support.**

17 **How interested are you in being a research partner in the future?**[ ] Very interested
[ ] Fairly interested
[ ] A little interested
[ ] Not at all interested
[ ] Other, please specify

18 **Please tell us more about what creates interest for you in being a research partner.**

19 **What could make it difficult for you to be a research partner? (Select all that apply)**

[ ] Health / energy
[ ] Vision, hearing, or mobility limitations
[ ] Time (e.g., other commitments)
[ ] Travel / transportation
[ ] Digital tools are difficult
[ ] Difficult language / 'research language'
[ ] Unclear role / expectations
[ ] I feel uncertain about what I can contribute
[ ] Previous negative experiences
[ ] No compensation for time / expenses
[ ] Other, please specify

20 **Please tell us more about how this makes it difficult for you to be a research partner.**

21 **What could make it easier for you to be a research partner? (Select all that apply)**

[ ] Clear information about the role and task
[ ] Adapted language and materials
[ ] Opportunity to participate in person if I need assistance with physical mobility
[ ] Opportunity to participate digitally from home
[ ] Help / support with technology
[ ] Compensation (e.g., honorarium, travel reimbursement)
[ ] Flexible times and shorter meetings
[ ] Opportunity to bring a support person / family member if needed
[ ] Feedback on how my views were used
[ ] Other, please specify

22 **Please tell us more about how this would make it easier for you to be a research partner.**

23 **If you were to be a research partner, in which parts of a study would you most like to contribute? (Select up to 3 options)**

[ ] Choosing important research questions
[ ] Planning how the study should be conducted
[ ] Developing information materials (letters, consent forms, advertisements)
[ ] Developing questionnaire / interview questions
[ ] Collecting data
[ ] Processing and analysis of data in interviews/ Investegations
[ ] Disseminating results (lectures, text, meetings)
[ ] Advisory role at different checkpoints
[ ] Do not know

24 **How would you prefer to participate?**

[ ] In-person meetings only
[ ] Digital participation only (e.g., Teams / Zoom)[ ] Both in person and digitally
[ ] Do not know

25 **What works best for you in terms of format and timing? (Select all that apply)**

[ ] Short meetings (60 minutes)
[ ] Longer meetings (90–120 minutes)
[ ] Daytime
[ ] Evening
[ ] Weekend
[ ] One-off occasions
[ ] Recurring involvement over a longer period

26 **How important is compensation for your ability to participate?**

[ ] Very important
[ ] Fairly important
[ ] A little important
[ ] Not important
[ ] Do not know

27 **Is there anything else that you think is important for older adults to be able to participate as research partners?**

Ageing, Meaningfulness, Participation, Financial Situation, and Digital Technology

The following questions concern how you experience ageing, your participation in society, your financial situation, and your use of digital technology.

Previous research has shown that such factors may influence the possibility of being active and involved in different contexts, such as research. Your responses help us better understand which conditions may promote or hinder older adults' participation as research partners.

28 **Below are a number of statements about ageing that we would like you to respond to.**

	Yes	No
Do you feel that life becomes worse the older you get?	o	o
Do you have as much energy now as you had one year ago?	o	o
Do you feel less needed the older you get?	o	o
Is life in older age better or worse than you had imagined?	o	o
Are you as happy now as you were when you were younger?	o	o

29 **Below are a number of statements about meaningfulness and participation that we would like you to respond to.**

Please indicate how much you agree or disagree with each statement, where 1 = Strongly disagree and 5 = Strongly agree.

	1	2	3	4	5
My life is meaningful.	o	o	o	o	o
I feel involved in society.	o	o	o	o	o
I feel valuable.	o	o	o	o	o

30 **Overall, how would you describe your current financial situation?**[ ] Very good
[ ] Fairly good
[ ] Neither good nor poor
[ ] Fairly poor
[ ] Very poor

31 **Is your financial situation sufficient for you to participate in the activities you would like to take part in?**
[ ] Yes
[ ] No

32 **How often do you use the internet?**[ ] Daily
[ ] A few times per week
[ ] A few times per month
[ ] Rarely / never

33 **Do you sometimes avoid using the internet because you feel insecure or unsafe?**[ ] Yes
[ ] No

34 **Below are a number of statements about technology and the internet that we would like you to respond to.**

Please indicate how much you agree or disagree with each statement, where 1 = Strongly disagree and 5 = Strongly agree.

	1	2	3	4	5
I think new technological devices are fun.	o	o	o	o	o

	1	2	3	4	5
I am sometimes afraid that I will not be able to use new technological devices.	o	o	o	o	o
Using technology makes life easier for me.	o	o	o	o	o
Today, technological development is moving so fast that I find it difficult to keep up.	o	o	o	o	o
I am happy to get the latest models or updates.	o	o	o	o	o
I would dare to try new technological devices to a greater extent if I had more support and help than I have today.	o	o	o	o	o

35

Below are a number of statements about digital technology.

Please indicate how much you agree or disagree with each statement, where 1 = Strongly disagree and 5 = Strongly agree.

	1	2	3	4	5
Digital technology helps me a great deal with practical tasks in everyday life.	o	o	o	o	o
Digital technology is an important part of my social life.	o	o	o	o	o
My smartphone is very useful to me in everyday life.	o	o	o	o	o
I often use the internet to obtain new knowledge.	o	o	o	o	o
I am among those who are usually first to adopt new digital technology.	o	o	o	o	o
It can be said that digital technology is an important part of my life.	o	o	o	o	o

36

If you wish: May we contact you about future opportunities to participate as a research partner?

[ ] Yes

[ ] No

37

If yes: Contact details (email / telephone)

Thank you for taking the time to respond. Your answers contribute to increased knowledge about how older adults can participate as co-researchers.
