# Supplementary material for: Strategies and enabling conditions for strengthening older adults’ involvement as active research partners: protocol for a sequential mixed-methods study in Sweden
Source: BMJ Open. 2026 Jul 20;16(7):e118308. doi: 10.1136/bmjopen-2026-118308 (PMC13386054; doi:10.1136/bmjopen-2026-118308)
Supplement: online supplemental file 2 [file bmjopen-16-7-s002.pdf]

Researchers' Experiences of Involving Older Adults as Active Research Partners

Appendix-ready English version of the questionnaire used in the study

Purpose: This questionnaire aims to map how researchers work with, experience, and view opportunities and barriers to involving older adults (60+) as active research partners in health and social care research.

A research partner refers to an older adult who is involved beyond the role of study participant, for example in planning, conduct, analysis, or dissemination of research.

Participation and confidentiality: Participation is voluntary. Responses are handled confidentially and reported only at group level.

More information: Full participant information about the study, including how data are handled and what rights participants have, is available in the study information sheet.

Estimated completion time: approximately 10–15 minutes.

The original questionnaire was administered in Swedish. This English version has been formatted as a clean publication appendix.

1 **I have read the information above and consent to participate.**

[ ] Yes

Background

2 **Main research area**

[ ] Medicine
[ ] Nursing
[ ] Social work
[ ] Public health
[ ] Gerontology/ageing
[ ] Rehabilitation
[ ] Other, please specify

____

3 **Number of years as an active researcher (after PhD or equivalent)**

[ ] < 5 years
[ ] 5–10 years
[ ] 11–20 years
[ ] > 20 years
[ ] Doctoral student / pre-doc

4 **Current institutional affiliation**

[ ] University / higher education institution
[ ] Research institute
[ ] Regional health care organization
[ ] Municipality
[ ] Company
[ ] Other, please specify

5 **Current role**

[ ] Doctoral student
[ ] Postdoctoral researcher
[ ] Associate Senior Lecturer
[ ] Senior Lecturer / Assistant Professor
[ ] Associate Professor / Docent
[ ] Professor
[ ] Clinical researcher
[ ] Other, please specify

Experience of Involving Older Adults as Research Partners

6 **Have you ever involved older adults (60+) as research partners in your research?**

[ ] Yes
[ ] No

If YES in Question 6, please answer Questions 7–9. If NO, please answer Questions 10–12.

7 **If yes: In which parts of the research process have older adults been involved? (Select all that apply)**

[ ] Identification of research questions
[ ] Development of study design / method
[ ] Development of information materials / consent materials
[ ] Data collection
[ ] Analysis / interpretation of results
[ ] Dissemination / implementation
[ ] Reference group / user advisory council
[ ] Other, please specify

8 **How would you assess these experiences overall?**

[ ] Very positive
[ ] Fairly positive
[ ] Mixed
[ ] Fairly negative
[ ] Very negative

9 **To what extent did you experience that the involvement contributed to the study's quality and relevance?**

[ ] To a very high degree
[ ] To a high degree
[ ] To some degree
[ ] To a low degree
[ ] Not at all

10 **What are the main reasons why you have not yet involved older adults as research partners? (Select all that apply)**

[ ] I have not had the opportunity within my projects
[ ] I lack knowledge about how involvement can be carried out in practice
[ ] I have lacked methodological support or guidance
[ ] Lack of time has been a barrier
[ ] There has been insufficient funding or resources
[ ] It has not been relevant to my research questions
[ ] I am uncertain how older adults could contribute
[ ] Ethical or legal uncertainties have been a barrier
[ ] The organization or leadership has not prioritized this type of involvement
[ ] I have not had contact with suitable older adults or networks
[ ] Other, please specify

____

11 **How open are you to involving older adults as research partners in future projects?**

[ ] Very open
[ ] Fairly open
[ ] Neither / nor
[ ] Fairly hesitant
[ ] Very hesitant

12 **Under what conditions would you consider involving older adults as research partners?**

Organizational Support, Training, and Methodological Readiness

13 **To what extent do you feel that your organization encourages the involvement of older adults as research partners?**

[ ] To a very high degree
[ ] To a high degree
[ ] To some degree
[ ] To a low degree
[ ] Not at all

14 **Have you had access to funding that enabled such involvement (e.g., honoraria, time, support)?**

[ ] Yes
[ ] No
[ ] Do not know

15 **Have you received any education or supervision in methods for user/patient involvement?**

[ ] Yes, formal training
[ ] Yes, informal (e.g., via colleagues/projects)
[ ] No

16 **How sufficient do you consider your own methodological competence to be when it comes to involving older adults as research partners?**

[ ] Very sufficient
[ ] Fairly sufficient
[ ] Neither / nor
[ ] Fairly insufficient
[ ] Very insufficient

Perceived Conditions for Involving Older Adults as Research Partners

17 **Please indicate the extent to which you agree with the following statements about individual conditions for involving older adults as active research partners.**

Response scale: 1 = Strongly disagree, 5 = Strongly agree.

	1	2	3	4	5
I have sufficient knowledge about how older adults can be involved as active research partners.	o	o	o	o	o
I feel confident working with older adults as co-researchers or collaborative partners.	o	o	o	o	o
I experience that involving older adult research partners enriches the research process.	o	o	o	o	o
I am uncertain about what role older adults should have in research projects.	o	o	o	o	o
I experience that involving older adults entails ethical or practical difficulties.	o	o	o	o	o

18 **Please indicate the extent to which you agree with the following statements about methodological and practical conditions for involving older adults as active research partners.**

Response scale: 1 = Strongly disagree, 5 = Strongly agree.

	1	2	3	4	5
I experience that there is sufficient methodological support for involving older adults as research partners.	o	o	o	o	o
The involvement of older adults can be adapted to different research designs.	o	o	o	o	o
Involving older adult research partners requires more time than is realistic in my projects.	o	o	o	o	o

	1	2	3	4	5
It is clear how older adults' contributions can be integrated into analysis and results.	o	o	o	o	o
It is difficult to find suitable older adults to involve in research.	o	o	o	o	o

19 **Please indicate the extent to which you agree with the following statements about organizational and structural conditions for involving older adults as active research partners.**

Response scale: 1 = Strongly disagree, 5 = Strongly agree.

	1	2	3	4	5
In practice, I experience that my organization provides active support for involving older adults as research partners.	o	o	o	o	o
There are sufficient resources (time, funding, support) to work with involvement.	o	o	o	o	o
The involvement of older adult research partners is valued in merit and assessment systems.	o	o	o	o	o
Administrative or legal requirements make it more difficult to involve older adults.	o	o	o	o	o
Leaders and project managers support work with research partnerships.	o	o	o	o	o

20 **Please indicate the extent to which you agree with the following statements about relational and cultural aspects of involving older adults as active research partners.**

Response scale: 1 = Strongly disagree, 5 = Strongly agree.

	1	2	3	4	5
Collaboration between researchers and older adult research partners is often characterized by equality.	o	o	o	o	o
Power imbalances between researchers and older adults are difficult to manage.	o	o	o	o	o
There is a risk of symbolic or tokenistic involvement of older adults.	o	o	o	o	o
Older adults' experiential knowledge is taken seriously in research contexts.	o	o	o	o	o
There is a research culture that supports co-creation with older adults.	o	o	o	o	o

21 **Please indicate the extent to which you agree with the following statements that concern your overall assessment of involving older adults as active research partners.**

Response scale: 1 = Strongly disagree, 5 = Strongly agree.

	1	2	3	4	5
Overall, I feel that the conditions for involving older adults as active research partners are good.	o	o	o	o	o
I would like to involve older adults more than I do today.	o	o	o	o	o
I see a need for more education or support regarding the involvement of older adult research partners.	o	o	o	o	o

Support Needs and Reflections

22 **What would make it easier for you to involve older adults as research partners to a greater extent in future projects? (Select all that apply)**

[ ] Clearer guidelines / frameworks
[ ] Methodological support and practical tools
[ ] Education / training
[ ] Institutional support (time, recognition)
[ ] Tailored funding opportunities
[ ] Administrative support
[ ] Access to established networks of older adult research partners
[ ] Other, please specify

23 **What type of support would you personally request the most?**

[ ] Methodological training
[ ] Practical examples / cases
[ ] Supervision / mentorship
[ ] Templates and checklists
[ ] Ethical support and guidance
[ ] None
[ ] Do not know
[ ] Other, please specify

24 **What does meaningful involvement of older adults as research partners mean to you as a researcher?**

25 **What risks do you see with involvement that is not meaningful?**

26 **Is there anything else you would like to share about involving older adults as research partners that has not been captured in the questions above?**

Optional Follow-up Questions

The following questions are entirely voluntary. Responses to these items should be handled separately from the main survey responses.

27 **Do you know of other researchers or research groups who may be interested in participating in this study?**

You may, for example, name a research group, institution, or organization. Please avoid sharing private contact details unless you have their consent.

28 **Do you know of older adults (60+) who might be interested in receiving information about the opportunity to participate in the study (e.g., in an interview or workshop)?**

[ ] Yes
[ ] No
[ ] Unsure

29 **If yes to the previous question: Please describe the type of context in which it might be appropriate to make contact (e.g., association, network, council).**

30 **Would you yourself consider participating in a follow-up interview about your experiences?**

[ ] Yes
[ ] No

31 **If yes: Please indicate how we can reach you. These details will be used only to contact you regarding an interview and will not be linked to your survey responses.**

Email address and telephone number (optional).

Thank you for taking the time to participate in the survey. Your experiences and reflections are valuable for the continued work of developing knowledge and support for involving older adults as research partners.
