# Supplementary material for: Strategies and enabling conditions for strengthening older adults’ involvement as active research partners: protocol for a sequential mixed-methods study in Sweden
Source: BMJ Open. 2026 Jul 20;16(7):e118308. doi: 10.1136/bmjopen-2026-118308 (PMC13386054; doi:10.1136/bmjopen-2026-118308)
Supplement: online supplemental file 3 [file bmjopen-16-7-s003.pdf]

Semi-structured interview guide for older adults

Older adults as active research partners

1. Background and context

Purpose: to understand the participant's experiences and point of departure

- Could you tell me a little about yourself and your interest in research?
- Have you previously been involved in any research project? If so, in what way?
- How did you first come into contact with research or researchers?

2. Experiences of involvement

Purpose: to capture concrete experiences

- Can you describe what your involvement looked like in practice?
- In which parts of the research process were you involved?
- What worked well, and what worked less well?
- Did you feel listened to and taken seriously? Why or why not?

3. Understanding research involvement

Purpose: to explore how older adults perceive the role of research partner

- What does it mean to you to be involved in research?
- How would you describe the difference between being a study participant and being a research partner?
- What kind of influence do you think it is important for older adults to have in research?

4. Conditions and barriers

Purpose: to identify enabling and limiting factors

- What do you think is needed for older adults to be meaningfully involved in research?
- Is there anything that may make it difficult to engage in research?
- How do you experience the support, information, and communication provided by researchers?

5. Meaningfulness, value, and motivation

Purpose: to understand what creates engagement and sustainable involvement

- What makes involvement feel meaningful to you?
- What could motivate more older adults to engage in research?
- Do you feel that your contribution can make a difference? In what way?

6. Future perspectives and development

Purpose: to capture participants' perspectives on improvement and future development

- How would you like older adults to be involved in research in the future?
- Is there anything researchers should do differently?
- Is there anything else you would like to add that we have not discussed?
