# Supplementary material for: Strategies and enabling conditions for strengthening older adults’ involvement as active research partners: protocol for a sequential mixed-methods study in Sweden
Source: BMJ Open. 2026 Jul 20;16(7):e118308. doi: 10.1136/bmjopen-2026-118308 (PMC13386054; doi:10.1136/bmjopen-2026-118308)
Supplement: online supplemental file 4 [file bmjopen-16-7-s004.pdf]

Semi-structured interview guide for researchers

Older adults as active research partners

1. Background and experience

Purpose: to understand the researcher's context and previous work

- Could you tell me about your research background and research area?
- Could you describe your experience of involving older adults as active research partners?

2. Practical implementation

Purpose: to illuminate how involvement is organized in practice

- How has the involvement of older adults been organized in practice in your projects?
- How are older adult research partners recruited?
- How have roles, responsibilities, and expectations been managed?

3. Views on older adults as research partners

Purpose: to explore the researcher's understanding and perspective

- How do you define the role of older adults in research?
- What does "meaningful involvement" mean to you as a researcher?
- In which parts of the research process do you see older adults contributing most?

4. Opportunities and challenges

Purpose: to identify structural, organizational, and relational factors

- What opportunities do you see in involving older adults as research partners?
- What challenges or barriers have you encountered?
- How do you address issues of power, representation, and inclusion?

5. Support structures and competencies

Purpose: to explore needs for organizational and methodological support

- What support have you had (or lacked) in working with involvement?
- Are there competencies or resources needed to improve this work?
- How do you view time, funding, and academic incentives in relation to involvement?

6. Learning and consequences

Purpose: to understand perceived effects of involvement

- In what ways has involving older adults influenced the research process or outcomes?
- Has it changed how you think about research?
- Is there anything you would do differently today?

7. Looking ahead

Purpose: to contribute to the project's developmental ambition

- What do you think is needed to strengthen the role of older adults as active research partners in the future?
- What structural changes are needed within research or organizations?
- Is there anything else you would like to add?
