# Supplementary material for: Strategies and enabling conditions for strengthening older adults’ involvement as active research partners: protocol for a sequential mixed-methods study in Sweden
Source: BMJ Open. 2026 Jul 20;16(7):e118308. doi: 10.1136/bmjopen-2026-118308 (PMC13386054; doi:10.1136/bmjopen-2026-118308)
Supplement: Supplementary file 5 [file bmjopen-2026-118308.reviewer_comments.pdf]

# PEER REVIEW HISTORY

BMJ Open publishes all reviews undertaken for accepted manuscripts. Reviewers are asked to complete a checklist review form and are provided with free text boxes to elaborate on their assessment. These free text comments are reproduced below.

## ARTICLE DETAILS

### Title (Provisional)

Strategies and enabling conditions for strengthening older adults' involvement as active research partners: protocol for a sequential mixed-methods study in Sweden

### Authors

Hedqvist, Ann-Therese; Strandberg, Susanna; Nilsen, Charlotta; Carlsson, Willemo; Carlsson, Roger; Violasdotter Nilsson, Paola; Holmberg, Mats; Ljungholm, Linda; Bergstrand, Sara; Andreassen, Maria; Holmberg, Bodil; Niklasson, Joakim

---

## VERSION 1 - REVIEW

---

| | |
|---|---|
| **Reviewer** | **1** |
| **Name** | **Chan Carusone, Soo** |
| **Affiliation** | **McMaster University, McMaster Collaborative for Health and Aging** |
| **Date** | **06-Apr-2026** |
| **COI** | **None** |

---

"Strengthening the Role of Older Adults as Active Research Partners: Protocol for a Mixed-Methods Study" is well-written and will hopefully help advance the practice and reporting of partnered research related to aging and older adults. Please see my specific comments and suggestions:

- Please report some dates for this work, for example when it was started and if any of the activities have set dates.

- I think it is important to emphasize the difference in research practices and societal perceptions of research across regions and cultures. While this is mentioned, it is unclear how this is considered in the study. I assume the umbrella review will include all systematic reviews (regardless of language, geographical scope, etc.) will you do any analysis based on region/country? And for the other phases, who will be included? Only older adults and researchers in Sweden? If so, all of Sweden? Is the goal to have the outcomes specific to Sweden? If so this should be made clear throughout the paper. (And if not, I am not sure the methods are appropriate to achieve statements that apply globally.)

- You mention the two older adults who were part of the team, I suggest providing some more detail about who else is on the team and perhaps how the team was assembled.

- In Figure 1, "integrate findings" appears after the Delphi process – should there not be an integration of findings in the exploratory phase to inform the questionnaire and Delphi process?

- Under "Participants and recruitment" please provide more details on the inclusion/exclusion of participants (including, as mentioned above, geographical restrictions, and language). Also, will the qualitative interview participants be drawn from the survey participants?

- I am unfamiliar with the term "PPI representatives" – suggest rewording this

- You state that "Researcher panel members will be recruited through academic networks, research centres, and professional associations." Will these panel members be different from participants in earlier phases? (Entirely or partially?)

- Figure 2, suggest renaming this "study timeline" as there are no participants involved in a number of the stages presented. Please also add "months" to the figure to represent the units across the top (not just having it in the figure caption).

- Under the "Mapping survey" section, I suggest rewording "potential confounders and key outcome measures" it is unclear what you mean – confounders to what? (for example in this sentence: The survey is designed to capture current practices, experiences, perceived barriers, and enabling conditions related to involving older adults as research partners, and includes three components for both groups: demographic variables, potential confounders, and key outcome measures.)

- On page 18/39, you state that the survey will capture "preferences for future involvement", do you mean in the later phases of this study, or preferences for being involved as a partner in research (in the future)?

- Please include what language(s) the survey will be available in

- Given the purpose of the interviews it makes sense to include only those with experience (as partners or working with older adult partners), is this the plan? Please make clear the inclusion criteria (and rationale).

- In the "Modified Delphi study" section you mention that "statements reaching predefined thresholds for high agreement … will be considered to reflect consensus." Are you analyzing separately by perspective (e.g., older adults and researchers separately)?

---

## VERSION 1 - AUTHOR RESPONSE

## Reviewer 1 comments

5. Please report some dates for this work, for example when it was started and if any of the activities have set dates.

**Response:**
We thank the reviewer for this suggestion. We have now added information on the study timeline, including the start date and planned timing of the different study phases. Details have been added in the Methods section and reflected in the study timeline figure.

6. I think it is important to emphasize the difference in research practices and societal perceptions of research across regions and cultures. While this is mentioned, it is unclear how this is considered in the study. I assume the umbrella review will include all systematic reviews (regardless of language, geographical scope, etc.) will you do any analysis based on region/country? And for the other phases, who will be included? Only older adults and researchers in Sweden? If so, all of Sweden? Is the goal to have the outcomes specific to Sweden? If so this should be made clear throughout the paper. (And if not, I am not sure the methods are appropriate to achieve statements that apply globally.)

**Response:**
We appreciate this important and insightful comment. The umbrella review includes international evidence, providing a broad conceptual understanding of involvement practices across contexts. The empirical phases (qualitative interviews, mapping survey, workshop, and Delphi study) are conducted primarily within a Swedish context, aiming to develop context-sensitive strategies. The intention is not to produce universally generalisable findings, but rather to contribute with empirically grounded insights that may be transferable to similar settings. Clarifications have been added in the study design, participants and recruitment, and discussion sections.

7. You mention the two older adults who were part of the team, I suggest providing some more detail about who else is on the team and perhaps how the team was assembled.

**Response:**
We thank the reviewer for this suggestion. We have expanded the description of the research team to provide additional information about its composition, expertise, and how the team was assembled. Additional details have been added in the Methods section, under Study Design.

8. In Figure 1, "integrate findings" appears after the Delphi process – should there not be an integration of findings in the exploratory phase to inform the questionnaire and Delphi process?

**Response:**
We agree with this observation. Figure 1 has been revised to reflect the iterative nature of the study design, including integration of findings during the exploratory phase to inform subsequent data collection steps. We have also updated Figure 2 to include this phase in the study timeline.

9. Under “Participants and recruitment” please provide more details on the inclusion/exclusion of participants (including, as mentioned above, geographical restrictions, and language). Also, will the qualitative interview participants be drawn from the survey participants?

**Response:**
We appreciate this comment and have expanded the description of inclusion and exclusion criteria, including geographical scope and language considerations. We have also clarified the relationship between the different samples, specifying that interview participants may be recruited independently or from survey respondents who indicate interest in further participation. Details have been added under respective section in Data collection.

10. I am unfamiliar with the term “PPI representatives” – suggest rewording this

**Response:**
Thank you for this comment. We have retained the term but clarified its meaning, as noted above, to improve transparency and accessibility for readers unfamiliar with this terminology. A definition has been added at first mention.

11. You state that “Researcher panel members will be recruited through academic networks, research centres, and professional associations.” Will these panel members be different from participants in earlier phases? (Entirely or partially?)

**Response:**
We have clarified that Delphi panel participants may partially overlap with participants from earlier phases, but that additional participants will also be recruited to ensure broader representation and diversity of perspectives. Clarification added in the Modified Delphi study section.

12. Figure 2, suggest renaming this “study timeline” as there are no participants involved in a number of the stages presented. Please also add “months” to the figure to represent the units across the top (not just having it in the figure caption).

**Response:**
We agree and have revised the figure accordingly. The figure has been renamed “Study timeline,” and months have been added to the x-axis to clarify the time units. Figure 2 has been updated.

13. Under the “Mapping survey” section, I suggest rewording “potential confounders and key outcome measures” it is unclear what you mean – confounders to what? (for example in this sentence: The survey is designed to capture current practices, experiences, perceived barriers, and enabling conditions related to involving older adults as research partners, and includes three components for both groups: demographic variables, potential confounders, and key outcome measures.)

**Response:**
We agree that this wording was unclear. As the survey is descriptive in nature, we have revised the terminology to better reflect the types of variables included. “Potential confounders” has been replaced with “background characteristics.”

14. On page 18/39, you state that the survey will capture “preferences for future involvement”, do you mean in the later phases of this study, or preferences for being involved as a partner in research (in the future)?

**Response:**
We thank the reviewer for this clarification request. We have revised the text to specify that this refers to preferences for future involvement as research partners in research more broadly.

15. Please include what language(s) the survey will be available in

**Response:**
We have now specified the language(s) in which the survey will be available. The information is added in the Mapping survey section.

16. Given the purpose of the interviews it makes sense to include only those with experience (as partners or working with older adult partners), is this the plan? Please make clear the inclusion criteria (and rationale).

**Response:**
We agree and have clarified that participants in the qualitative interviews will be selected based on prior experience of involvement as research partners or collaboration with such partners. The rationale for this approach has also been added. Clarification added in the qualitative interviews section.

17. In the “Modified Delphi study” section you mention that “statements reaching predefined thresholds for high agreement ... will be considered to reflect consensus.” Are you analyzing separately by perspective (e.g., older adults and researchers separately)?

**Response:**
We appreciate this important point. We have clarified that responses will be analysed both across the full sample and, where relevant, stratified by stakeholder group to explore potential differences in perspectives. Clarification added in the Modified Delphi study section.

---

## VERSION 2 - REVIEW

---

| | |
|---|---|
| **Reviewer** | **1** |
| **Name** | **Chan Carusone, Soo** |
| **Affiliation** | **McMaster University, McMaster Collaborative for Health and Aging** |
| **Date** | **03-Jul-2026** |
| **COI** | |

---

The edits are clear and address my concerns.
